# HPV Vaccine School Entry Requirement in Puerto Rico: Historical Context, Challenges, and Opportunities

**DOI:** 10.5888/pcd18.210035

**Published:** 2021-08-05

**Authors:** Vivian Colón-López, Coralia Vázquez-Otero, Vilnery Rivera-Figueroa, Glizette O. Arroyo-Morales, Diana T. Medina-Laabes, Roxana Soto-Abreu, Olga L. Díaz-Miranda, Ángel Rivera, Iris Cardona, Ana P. Ortiz, VOCESPR, Pamela C. Hull

**Affiliations:** 1 Puerto Rico Cancer Control and Population Sciences Division, University of Puerto Rico Comprehensive Cancer Center, San Juan, Puerto Rico; 2 Evaluation Research of Health Systems Science Program, Department of Health Services Administration, School of Public Health, Medical Science Campus, University of Puerto Rico, San Juan, Puerto Rico; 3 Harvard T.H. Chan School of Public Health & Dana-Farber Cancer Institute, Boston, Massachusetts; 4 Department of Biostatistics and Epidemiology, Graduate School of Public Health, Medical Sciences Campus, University of Puerto Rico, San Juan, Puerto Rico; 5 Puerto Rico Department of Health, San Juan, Puerto Rico; 6 VOCESPR, Coalición de Inmunización y Promoción de la Salud de Puerto Rico, Guaynabo, Puerto Rico; 7 Department of Behavioral Science, University of Kentucky, College of Medicine, Markey Cancer Center, Lexington, Kentucky

Epidemiologic studies in Puerto Rico have documented the prevalence of human papillomavirus (HPV) infection on the island, reporting rates up to 79% (1). Regarding HPV-related cancers, Puerto Rico has the highest incidence rate of cervical cancer among all the states and territories of the United States (2). Despite the lower prevalence of anal cancer in Puerto Rico, men who have sex with men (MSM), people living with HIV, and women with HPV-related gynecological cancers are at higher risk of developing this malignancy (1,2). Moreover, the incidence of penile cancer is twice as high, and the mortality rate is 3 times as high among Puerto Rican men compared with men from other racial and ethnic groups in the US (3).

## Paving the Way for the Policy: Grassroots Movements and Other Organizations

Parallel with these scientific efforts to estimate the impact of HPV in Puerto Rico, grassroots movements have promoted educational campaigns and outreach activities to increase HPV vaccine uptake and awareness at the community level, as well as developed training opportunities among health care professionals. These efforts from multiple sectors (eg, coalitions, government agencies, health providers, scientists) led Puerto Rico to have among the highest HPV initiation rates since 2014. These sectors have been pioneers in developing policies that support HPV vaccination access in Puerto Rico. Among the more meaningful is Law No. 9 (passed December 20, 2010), which provided HPV vaccine access to girls aged 11 to 18 years. The law was amended in 2012 to also include boys aged 11 to 18 years (4). Implementation of this public policy was essential because the Vaccine for Children Program, which is available for Puerto Rican families with no health insurance, provides limited access to the vaccine for children from families with private insurance. In 2015, after the implementation of this law, the Vaccination Coalition of Puerto Rico (VOCES, by its acronym in Spanish) led the first discussion about having an HPV vaccine requirement for school entrance, as part of the HPV Advisory Panel report (5).

In Puerto Rico, under Law No. 25 (passed September 25, 1983), the Puerto Rico Secretary of Health can decide which vaccines will be required for school entrance (Figure). The law states that no student or preschool child may be admitted or enrolled in a school or day care center if he or she is not properly immunized (4,5). In June 2017 via a press conference, the Puerto Rico Secretary of Health encouraged parents and legal guardians to vaccinate their children against HPV and notified them that the vaccine was going to be required for school entrance for children aged 11 to 12 years starting in the fall of 2018. In May 2018, the official requirement was announced for implementation in August of 2018 (5).

**Figure:**
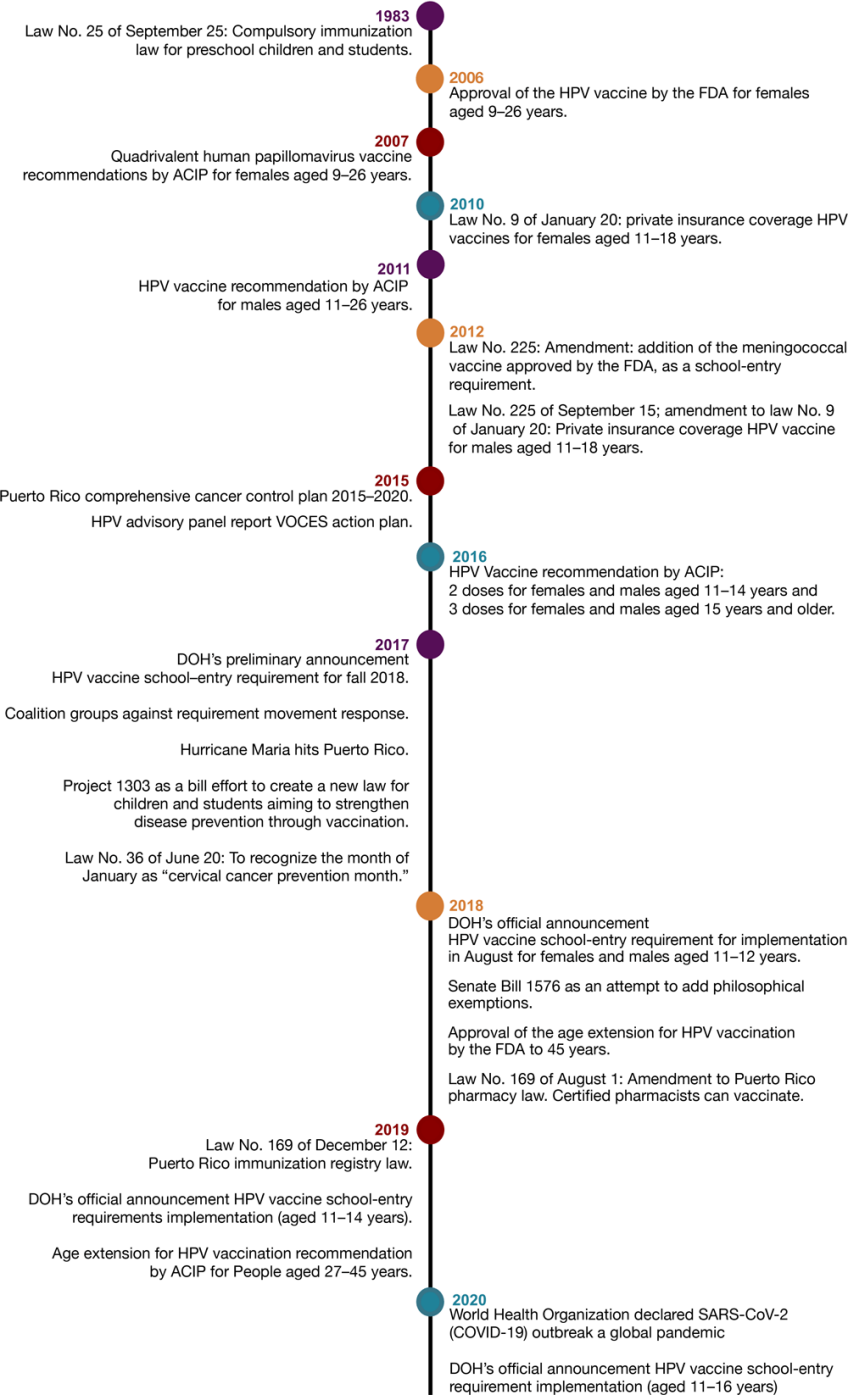
Timeline of events from adoption to implementation of the human papillomavirus (HPV) vaccine school-entry requirement in Puerto Rico, 1983–2020. Abbreviations: ACIP, Advisory Committee on Immunization Practices; DOH, Puerto Rico Department of Health; FDA, Federal Drug Administration; SARS-CoV-2, severe acute respiratory syndrome coronavirus 2; VOCES, Coalición de Vacunación de Puerto Rico.

## First 2 Years of The New School-Entry Requirement

Notably, there was a 1-year gap between the public notification and the policy adoption. Given this time frame, several groups voiced their concerns against the mandate (although not necessarily against the HPV vaccine) and publicly expressed their apprehension about the new requirement. Research has shown that this period permitted the development of bills intended to deter or “water down” the new requirement (eg, intended to include a philosophical exemption as part of Law No. 25) (5).

Adopting this policy took many years and much groundwork (eg, legislation, education) to accomplish. Moreover, the death of a young mother of 3 from cervical cancer in 2015 catalyzed a movement led by VOCES to advance the work surrounding HPV vaccination (5). The epidemiologic impact of the disease was considered before the policy’s adoption, as were the high HPV vaccine initiation rates. In 2016, according to the National Immunization Survey–Teen, rates before the implementation of the requirement were 80.8% in girls and 71.1% in boys with 1 or more HPV vaccine doses (6). Another consideration for the adoption was the initial cohort chosen (ie, children aged 11 to 12 years), which requires only 2 doses of the vaccine, resulting in a more cost-efficient approach (5).

Our team conducted key informant interviews with implementers (eg, school staff, health providers, coalitions and community leaders). We also interviewed participants who were opposed to the policy to explore the factors that facilitated or hindered the successful implementation of the HPV vaccine school-entry requirement in Puerto Rico. The principal barriers identified were misinformation about the HPV vaccine and lack of knowledge about the implementation process of the mandate. In contrast, people’s principal concern against the policy was the government’s excessive interference in deciding what was best for their children. During this activity, the key informants’ recommendations were to 1) inform parents about the pros and cons of the vaccine; 2) actively use social media as a channel for dissemination of reliable information, acknowledging how this media is also used to spread misinformation; 3) train school staff; and 4) identify a central-figure school staff member who can follow up on the completion of doses and guide the parents. Additional findings indicated parental hesitancy, lack of awareness about the HPV vaccine and the new school-entry vaccination requirement, and unfounded concerns about the potential side effects of the HPV vaccine (7).

Preliminary analyses using data from the Puerto Rico Immunization Registry (PRIR) showed an increase of 54.0% in vaccinations among children aged 11 to 12 years when comparing the pre-policy (2017) and post-policy (2019) periods. Nevertheless, several challenges are worth discussing. First, HPV vaccine implementation was 11 months after Hurricane Maria, one of the worst natural disasters in Puerto Rican history. The impact of this hurricane on the school and public health systems led to an estimated 2.43% population migration to the continental US in 2017 (approximately 77,000 people) (8). This migration dynamic should be carefully explored when calculating immunization rates using data from PRIR. Also, the impact of social media on hesitancy to use the HPV vaccine is worth documenting. Negative sentiments toward the vaccine may affect people’s intention to vaccinate, thus limiting improvements in vaccination rates (9). Additionally, the COVID-19 pandemic has altered the public health agenda, prioritizing efforts on mitigating this infection and implementing a sound COVID-19 vaccine distribution plan island wide. As expected, an impending and long-term challenge will be the impact COVID-19 has on adolescents’ vaccination use, as declines in doctor’s visits have been documented (10).

## New Opportunities

The recent implementation of policy that establishes an HPV vaccine requirement for school entry in Puerto Rico provides a unique opportunity to assess the impact on HPV vaccination rates on the island and to explore the broader context of HPV vaccine school-entry requirements in other US states and territories. This implementation also encourages the strengthening of partnerships between local communities and policy makers to create additional strategies to support this requirement and address challenges. Educational programs about HPV vaccination must be disseminated to reduce public skepticism and clarify misinformation among hesitant parents. Furthermore, epidemiologic studies should be developed to assess the impact of changes in HPV vaccination rates on future incidence of HPV-related precancers and cancers in Puerto Rico, with the potential to document the progress toward eliminating cervical cancer on the island.
